# Perception of fall risk among cancer survivors receiving Radiotherapy/Chemotherapy in China: A latent profile analysis

**DOI:** 10.1371/journal.pone.0358845

**Published:** 2026-09-24

**Authors:** Hongkun Liu, Yuping Cheng, Lin Wang, Hanfei Cui, Xuebing Jing, Shanshan Zhang

**Affiliations:** 1 Integrated Chinese and Western Medicine Orthopedics Department, Zibo Central Hospital, Zibo, China; 2 Interventional Oncology, Zibo Central Hospital, Zibo, China; 3 Clinical Research Center, Zibo Central Hospital, Zibo, China; 4 Nursing Department, Zibo Central Hospital, Zibo, China; Lamar University, UNITED STATES OF AMERICA

## Abstract

**Background:**

Accurate perception of fall risk is imperative for functional rehabilitation and quality of life among cancer survivors receiving radiotherapy/chemotherapy; however, current levels of awareness remain suboptimal. This study aimed to investigate the current status of fall risk perception among this population in China, identify its latent profiles and associated factors, and establish a theoretical basis for targeted nursing interventions.

**Methods:**

Convenience sampling was conducted. A total of 534 cancer survivors undergoing radiotherapy/chemotherapy were recruited from a tertiary hospital in Zibo City between June and September 2025. The study employed a general information questionnaire, the Fall Risk Perception Scale, the Social Support Rating Scale, the Health Literacy Management Scale, and the Self-Efficacy for Managing Chronic Disease Scale. Latent profile analysis (LPA) was employed to identify heterogeneous subgroups based on perceived fall risk, and logistic regression analysis was subsequently used to examine factors associated with subgroup membership.

**Results:**

Among the 534 enrolled cancer survivors, LPA classified perceived fall risk into three distinct categories: the Low-level Perceptually Deficient Group (22.85%), the Medium-level Condition-dependent Group (49.25%), and the High-level Self-awareness Group (27.90%). Multivariate analysis identified several modifiable factors significantly associated (*P* < 0.05) with these profiles. These factors were categorized as physiological health factors (duration of illness, tumor stage, hypertension, fall history, and vision), psychological and behavioral factors (self-assessment of health status, self-efficacy, and health literacy), and social and environmental factors (marital status, education level, caregiver, social support, and use of walking aids).

**Conclusion:**

Cancer survivors undergoing radiotherapy/chemotherapy demonstrated an above-average perception of fall risk, with significant heterogeneity observed. Healthcare providers should implement personalized interventions tailored to the distinct fall risk perception characteristics of different subgroups to enhance fall risk perception and ultimately reduce the incidence of falls.

## Introduction

Malignant tumors represent a major global public health issue that seriously threatens human health. According to data released by the WHO International Agency for Research on Cancer, the number of new cancer cases worldwide reached 19.97 million in 2022, with China accounting for 24.15% of these cases [1]. The widespread implementation of early cancer screening technologies and the continuous optimization of comprehensive treatment regimens—including surgery, chemotherapy, radiotherapy, targeted therapy, and immunotherapy—have significantly improved survival rates among patients with cancer [2]. However, this increased survivorship has also drawn attention to other health challenges, such as the risk of falls [3]. Meta-analysis findings (e.g., Bird et al.) [4] indicate that the incidence of falls among patients with cancer is 15–16 times higher than that in the general population. This elevated risk substantially exceeds the global average fall incidence of 6.26% in the general population [5], particularly among those undergoing radiotherapy/chemotherapy. Studies have reported that 22%–37% of these cancer survivors experience at least one fall annually [6,7], and this figure can increase to 50% among patients with advanced-stage disease. More than 35% of patients who experience a fall sustain injuries of varying severity, which may even result in death. Fall-related injuries significantly increase hospitalization costs and prolong hospital stays. A systematic review demonstrated that the incidence of falls among adult cancer survivors is 23.0% (95% CI: 0.23, 0.24) [8]. This incidence is influenced by multiple factors, including age, sex, educational level, fall history, fear of falling, tumor stage, tumor type, radiotherapy/chemotherapy, polypharmacy, malnutrition, low body weight, cognitive impairment, and balance disorders.

Current evidence indicates that most cancer survivors have a markedly inadequate perception of their own fall risk. A study by Xie Yue et al. [9] involving 378 cancer survivors found that patients’ fall risk perception was only at a moderate level. Some patients did not adequately recognize their risk of falling, resulting in a considerable discrepancy between their subjective perception and actual fall risk. Similarly, Hoke et al. [10] conducted interviews with both nurses and patients, revealing generally low awareness of fall risk among cancer survivors. Even after receiving fall prevention education, some patients tended to overestimate their functional abilities in the hospital setting and failed to recognize that fall risk may fluctuate with changes in their medical condition [11]. Internationally, fall reduction is recognized as one of the six core safety goals of the International Patient Safety Goals. In China, the Standards for the Evaluation of Tertiary Hospitals (2022 Edition) have incorporated the prevention and reduction of patient falls as a key evaluation indicator [12]. Therefore, although falls pose a serious threat to cancer survivors and have received considerable global attention, survivors themselves continue to underestimate this risk.

Risk perception was originally proposed by Professor Bauer of Harvard University in 1960 to describe the influence of consumers’ perceived risks on their behaviors [13]. In 1990, American scholar Balock [14] first introduced the concept of risk perception into the field of health and disease. Hay [15] defined risk perception as an individual’s belief regarding the likelihood and severity of acquiring a disease or experiencing adverse outcomes. Brewer et al. [16] provided a more comprehensive interpretation, proposing that risk perception primarily comprises three dimensions: perceived likelihood, perceived susceptibility, and perceived severity. Building on these foundational concepts, Chinese scholar Nie [17] defined fall risk perception as a patient’s subjective judgment of the probability and potential severity of a fall during daily activities, as well as its perceived impact on physical, psychological, and social well-being. Meanwhile, cancer-related fatigue—a core symptom emphasized in the ESMO guidelines [18]—can significantly impair patients’ activity tolerance and alertness, thereby further increasing their risk of falls. Radiotherapy and chemotherapy can directly impair balance, muscle strength, and neurological function, making cancer survivors a high-risk population for falls. Consequently, fall risk perception in this population is not merely a matter of daily safety but also a critical factor influencing rehabilitation outcomes, long-term survival, and quality of life [18]. Studies have further indicated that the accuracy of risk perception directly influences patients’ adoption of effective preventive behaviors [19]. Approximately one-fourth to one-third of in-hospital falls are preventable. Improving patients’ awareness of fall risk promotes proactive preventive behaviors and may reduce fall incidence by up to 50%. A randomized controlled trial involving hospitalized cancer survivors demonstrated that individualized education delivered through both oral and written formats significantly improved fall risk perception [20]. This approach translates objective assessments (e.g., gait tests and medication reviews) into understandable information and compares them with patients’ subjective perceptions, thereby helping to correct cognitive biases. Therefore, integrating structured assessment of fall risk perception into routine clinical evaluations is recommended to facilitate targeted education and enhance fall prevention.

Current research on fall risk perception predominantly focuses on community-dwelling older adults, hospitalized patients, and individuals with chronic diseases, examining areas such as risk awareness, associated factors, scale development, and qualitative perspectives [21,22]. Among patients undergoing radiotherapy or chemotherapy, research remains largely limited to the development and validation of assessment tools [23]. While existing evidence highlights the role of risk perception in fall prevention and quality of life, substantial gaps remain in both understanding and clinical implementation. Key limitations include the following [24–26]. Firstly, most studies employ a single methodological approach. Although quantitative research can objectively identify associated factors and population characteristics, it often fails to reveal the underlying motivations and behavioral mechanisms that shape risk perception [24]. Secondly, the research perspective is predominantly based on the biomedical model. Despite the identification of numerous objective risk factors, little is known about how psychosocial factors—such as health literacy, self-efficacy, and social support—interact to influence fall risk perception [25]. Although social support is known to improve psychological well-being and self-efficacy, the mechanisms through which it influences fall risk perception remain unclear. Thirdly, the relationships among key psychological factors, including self-efficacy, health literacy, and fall risk perception, have not been fully elucidated [26]. There is a lack of integrated analysis examining how social support, health literacy, and self-efficacy jointly influence risk perception, as well as limited understanding of the personalized needs of patient subgroups with distinct risk profiles.

The Health Belief Model (HBM) was proposed by Hochbaum in the 1950s to explain and predict health behaviors from the patient’s perspective [27]. The model posits that an individual’s perception of health risks is central to motivating behavioral change. According to the HBM, individuals are more likely to adopt health-promoting behaviors when they believe they are susceptible to a health threat, perceive the threat as serious, and believe that preventive actions are both beneficial and feasible (see S1 Fig) [28]. Key constructs of the HBM include perceived susceptibility, perceived severity, perceived benefits, perceived barriers, self-efficacy, and cues to action. Among these constructs, perceived susceptibility and perceived severity jointly drive motivation to act, whereas self-efficacy influences an individual’s confidence and persistence in maintaining such behaviors. Ahn et al. [29] demonstrated that HBM-based educational interventions significantly enhanced self-efficacy among hospitalized patients, leading to greater engagement in osteoporosis prevention and fall prevention behaviors. Similarly, Vincenzo et al. [30] highlighted that ongoing reminders from family members, healthcare providers, and the community serve as effective cues to action, facilitating the transition from intention to behavior. Guided by the HBM, this study systematically examined factors influencing fall risk perception among cancer survivors across three domains: physical health, psychological and behavioral characteristics, and social and environmental context. This framework provides a theoretical foundation for developing tailored fall prevention strategies in clinical settings.

Latent profile analysis (LPA) is a person-centered statistical approach that classifies individuals into distinct latent subgroups based on observed indicators, thereby effectively capturing population heterogeneity [31]. In research on fall risk perception, LPA offers unique advantages by identifying subpopulations with distinct risk perception profiles and revealing underlying heterogeneity patterns. This approach supports the development of stratified and targeted interventions. By innovatively applying LPA, this study systematically examined the latent profiles and associated factors of fall risk perception among patients undergoing radiotherapy/chemotherapy. The findings are expected to provide empirical evidence for personalized management strategies and optimized clinical screening, facilitating a transition from population-wide interventions to precision interventions.

## Methods

### Study design

This study employed convenience sampling to recruit cancer survivors receiving radiotherapy/chemotherapy at a tertiary hospital in Zibo City between June and September 2025. The study was approved by the Medical Ethics Committee of Zibo Central Hospital (No.2025181) and was conducted in accordance with the Helsinki Declaration and its later amendments or comparable ethical standards. All participants provided written informed consent.Based on the sample size estimation method for analytical cross-sectional studies, the required sample size was at least 10 times the number of independent variables. Given the 35 independent variables included in this study, the minimum required sample size was 350 [32]. After accounting for a 10% attrition rate, the required sample size increased to at least 385 participants. Ultimately, a total of 534 patients were included.

### Participants

A total of 23 variables constitute the general demographic information, covering items such as gender and age.A total of 534 cancer survivors undergoing radiotherapy/chemotherapy were enrolled. The mean age was 60.12 ± 13.67 years, with 246 males (46.1%) and 288 females (53.9%). Detailed information is provided in Table 1.

**Table 1:** General information of the study subjects (N = 534).

Variable	Group	N (%)
**Age (years)**	<45	149（27.9%）
	45-60	197（36.9%）
	>60	188（35.2%）
**Sex**	Male	246（46.1%）
	Female	288（53.9%）
**Employment status**	Unemployed	75（14%）
	Employed	194（36.4%）
	On leave	47（8.8%）
	Retired	218（40.8%）
**Duration of illness (years)**	<3	264（49.4%）
	3-5	196（36.7%）
	>5	74（13.9%）
**Marital status**	Single	90（16.9%）
	Married	248（46.4%）
	Divorced/Widowed	196（36.7%）
**Monthly income (RMB)**	<5000	225（42.1%）
	5000-10000	196 (36.7%)
	>10000	113 (21.2%)
**Treatment modality**	Chemotherapy	98（18.4%）
	Radiotherapy	318（59.6%）
	Both	118（22.0%）
**Residence**	Rural	167（31.3%）
	Town	151（28.3%）
	Urban	216（40.4%）
**Education level**	High school	133（24.9%）
	College	165（30.9%）
	Bachelor’s	175（32.8%）
	Graduate	61（11.4%）
**Tumor stage**	Ⅰ	169（31.6%）
	Ⅱ	204（38.2%）
	Ⅲ	86（16.1%）
	Ⅳ	75（14.1%）
**Type of tumor**	Intestinal tumors	150 (28.1%)
	Lung tumors	80 (15.0%)
	Breast tumors	65 (12.2%)
	Stomach tumors	55 (10.3%)
	Esophageal tumors	50 (9.4%)
	Hematological malignancies	35 (9.6%)
	Nasopharyngeal tumors	30 (5.6%)
	Liver cancer	25 (4.7%)
	Prostate cancer	15 (2.8%)
	Kidney cancer	10 (1.9%)
	Laryngeal tumors	8 (1.5%)
	Oral tumors	6 (1.1%)
	Bladder cancer	3 (0.6%)
	Malignant brain tumor	1 (0.2%)
	Chondrosarcoma	1 (0.2%)
**Alcohol use**	Yes	197 (36.9%)
	No	337（63.1%）
**Smoking history**	Yes	143（26.8%）
	No	391（73.2%）
**Hypertension**	Yes	341（63.9%）
	No	193（36.1%）
**Diabetes**	Yes	133（24.9%）
	No	401（75.1%）
**Fall history**	Yes	142（26.6%）
	No	392（73.4%）
**Walker**	Use	139（26%）
	Nonuse	395（74%）
**Self assessment of health status**	Bad	100（18.7%）
	Commonly	238（44.6%）
	Good	196 (36.7%)
**Fear of falling**	Yes	347（65%）
	No	187（35%）
**Exercise**	Hobby	256（47.9%）
	Not hobby	278（52.1%）
**Caregiver**	Family	253（47.4%）
	Nursing workers	158（29.6%）
	No caregiver	123（23%）
**Vision**	Normal vision	239（44.8%）
	Vision disorder	295（55.2%）
**Sleep**	Insomnia	186（34.8%）
	Difficulty falling asleep	145（27.2%）
	Help with sleep	120（22.5%）
	Sleep well	83（15.5%）

### Inclusion and exclusion criteria

The inclusion criteria were as follows: (1) age ≥ 18 years; (2) pathologically confirmed malignant tumor; (3) hospitalized patients receiving radiotherapy, chemotherapy, or both; (4) expected survival of ≥ 6 months; and (5) willingness to participate and provision of written informed consent.

The exclusion criteria were as follows: (1) a history of mental illness or cognitive impairment; (2) severe complications; and (3) inability to complete the questionnaire.

### Research Tools

#### Fall risk perception scale (FRPS).

This instrument was developed to assess cancer patients’ perception of fall risk and was compiled in 2025 by the Chinese scholars Luo Ruijun et al. [33]. The scale includes five dimensions: fall susceptibility perception (5 items), physiological condition susceptibility (12 items), personal activity susceptibility (5 items), environmental factor susceptibility (5 items), and fall severity perception (3 items), comprising a total of 30 items. The scale uses a 5-point Likert scoring system, with positively worded items scored from 1 (“strongly disagree”) to 5 (“strongly agree”). Total scores range from 30 to 150, with higher scores indicating better fall risk perception. The scale demonstrated high reliability, with a reported overall Cronbach’s α of 0.926 and values ranging from 0.914 to 0.970 across its dimensions [33]. In the present study, the Cronbach’s α coefficient was 0.843.

#### Social support rating scale (SSRS).

The SSRS developed by Shuiyuan [34] in 1986, was used to evaluate individuals’ levels of social support. It includes three dimensions: objective support, subjective support, and support utilization, comprising a total of 10 items. Items 1–4 and 8–10 are scored using a 4-point Likert scale, whereas Item 5 is scored from 1 (“no support”) to 4 (“full support”), and Items 6 and 7 are scored according to the number of support sources, with “no sources” scored as 0 points. Total scores range from 12 to 66, with higher scores indicating stronger social support. Scores are categorized as high (45–66), moderate (23–44), or low (≤22). The scale demonstrated excellent reliability, with a Cronbach’s α coefficient of 0.920 and test–retest reliability ranging from 0.890 to 0.940 [34]. In the present study, the Cronbach’s α coefficient for the SSRS was 0.916.

#### Health literacy management Scale(HLMS).

The HLMS was originally developed by Jordan et al. [35] and was later adapted into Chinese by Sun Haolin in 2012 [36].This scale was designed to assess the health literacy level of patients with chronic diseases, defined as the ability of individuals to acquire, understand, and apply health information to maintain and promote their own health.It contains 24 items across four dimensions: information access ability (9 items), communication and interaction ability (9 items), willingness to improve health (4 items), and willingness to provide economic support (2 items). Responses are measured using a 5-point Likert scale ranging from 1 (“very difficult”) to 5 (“very easy”). Total scores range from 24 to 120, with higher scores indicating better health literacy. The scale demonstrated a Cronbach’s α coefficient of 0.91 [36], whereas the Cronbach’s α coefficient in the present study was 0.876.

#### Self-efficacy to manage chronic disease scale (SEMCD).

The SEMCD was originally developed by the Stanford University Center for Patient Education [37] and was later simplified by Lorig et al. into a 6-item version (SEMCD-6) [38]. The scale is designed to assess patients’ confidence in managing recent health-related challenges, including overcoming fatigue, pain management, emotional regulation, symptom control, activity management, and medication adherence. The first four items assess self-efficacy in symptom management, whereas the last two assess self-efficacy in managing general aspects of chronic disease. Each item is scored on a 10-point scale ranging from “completely unconfident” to “completely confident.” The total score is calculated as the average of all items and is categorized as low (≤4.0), moderate (4.0–7.9), or high (≥8.0). The scale demonstrated strong reliability, with Cronbach’s α values ranging from 0.88 to 0.95 [38]. In the present study, the Cronbach’s α coefficient was 0.912.

### Data collection

The research team comprised four head nurses, two nursing team leaders, and two charge nurses from the oncology department. All team members received standardized training on participant recruitment and survey administration procedures and passed a qualification assessment. Two designated nurses recruited eligible participants from four oncology wards, obtained informed consent, and administered the survey using paper-based questionnaires. The estimated completion time was approximately 15–20 minutes. Participants completed the questionnaires independently. For those with limited literacy or other difficulties, researchers provided assistance by recording responses verbatim. A total of 560 questionnaires were distributed, and 534 valid questionnaires were returned, yielding an effective response rate of 95.36%. This study implemented on-site quality control throughout the data collection process. Each completed questionnaire was reviewed by the investigator immediately upon receipt, and participants were promptly asked to complete any missing responses.

### Statistical analysis

Mplus version 8.3 was used to perform LPA, with fall risk perception scores（FRPS） serving as the observed indicators. Model estimation began with a one-class model (C1), and the number of latent classes was increased sequentially until the optimal model fit was identified. Model fit was evaluated using the Akaike Information Criterion (AIC), Bayesian Information Criterion (BIC), and sample-size-adjusted BIC (aBIC), with lower values indicating better model fit. Entropy was used to assess classification accuracy, with values closer to 1 indicating higher classification precision; values ≥ 0.80 generally indicate classification accuracy exceeding 90%. The Lo–Mendell–Rubin adjusted likelihood ratio test (LMR) and bootstrap likelihood ratio test (BLRT) were used to compare competing models. A *P* value < 0.05 indicated that the K-class model provided a significantly better fit than the K − 1 class model. In addition, classification accuracy was evaluated by calculating the average posterior probability of assignment for each latent class, with values ≥ 0.80 generally considered indicative of good classification accuracy. SPSS version 26.0 was used for statistical analyses. Categorical variables collected via the general information questionnaire (e.g., age group, sex, education level, tumor stage, fall history, hypertension, caregiver status) are presented as frequencies and percentages. Continuous variables, including scores on the FRPS, SSRS, HLMS, and SEMCD, are expressed as means and standard deviations (Mean ± SD). Based on the optimal latent profile model, chi-square tests and one-way analysis of variance were used to compare differences among latent classes in general characteristics, fall risk perception, health literacy, self-efficacy, and social support. Specifically, chi-square tests were employed for categorical demographic and clinical variables, while one-way ANOVA was used for continuous scale scores (SSRS, HLMS, and SEMCD total scores). The variance inflation factor (VIF) and tolerance values were used to assess multicollinearity among all independent variables. VIF values < 5 or tolerance values > 0.20 were considered indicative of the absence of significant multicollinearity. Multivariate logistic regression analysis was performed to identify factors associated with latent profile membership, with the three latent classes as the dependent variable (using the Low-level Perceptually Deficient Group as the reference category) and factors statistically significant in univariate analyses as independent variables. A P value < 0.05 was considered statistically significant.

## Results

A total of 560 questionnaires were distributed in this study. Following rigorous quality control procedures, 26 invalid questionnaires were excluded based on predefined criteria: 15 due to logical inconsistencies, 8 due to patterned responses, and 3 due to completion times of less than 3 minutes. The final sample comprised 534 patients aged 35–79 years (mean age: 60.12 ± 13.67 years), including 246 (46.1%) men and 288 (53.9%) women. Detailed demographic and clinical characteristics are presented in Table 1.

### Results of LPA

An exploratory LPA of the FRPS was conducted using one- to five-class models (Table 2). As the number of latent classes increased, the AIC and aBIC values consistently decreased. All models demonstrated entropy values > 0.80, indicating good classification quality. After considering all fit indices and clinical interpretability, the three-class model was selected as the optimal solution. This model exhibited an entropy value of 0.990, and both the LMR and BLRT were statistically significant (*P* < 0.01). In contrast, the four-class model contained a subgroup comprising only 4.9% of the sample, whereas the five-class model failed to demonstrate a significant BLRT result (*P* = 0.05), indicating inadequate model parsimony and stability. Based on item-level scores from the FRPS, the three latent profiles of fall risk perception among cancer survivors are presented in S2 Fig. The average posterior assignment probabilities for the three latent classes were 1.000, 1.000, and 0.993, respectively, all substantially exceeding the recommended threshold of 0.80. These findings indicate that the LPA model achieved excellent classification accuracy and clear discrimination among the latent classes.

**Table 2:** Fit Statistics for the Latent profile analysis (N = 534).

Model	AIC	BIC	ABIC	LMRT	BLRT	Entropy	Class Probabilities	Classification accuracy
**1**	17816.640	17859.444	17827.700	−	−	−	−	1.000
**2**	16003.631	16072.117	16021.328	0.0000	0.0000	1.000	0.50749/0.49251	1.000
**3**	15107.764	15201.933	15132.098	0.0000	0.0000	0.990	0.22846/0.49251/0.27903	0.993
**4**	14673.575	14793.426	14704.545	0.0000	0.0000	0.990	0.04869/0.49251/0.22846/0.23034	0.966
**5**	14372.204	14517.738	14409.811	0.0500	0.0000	0.981	0.22846/ 0.04869/0.37640/0.23034/ 0.11610	0.957

Note: AIC, Akaike Information Criterion; BIC, Bayesian Information Criterion; aBIC, adjusted Bayesian Information Criterion; LMR, Lo–Mendell–Rubin adjusted likelihood ratio test; BLRT, bootstrap likelihood ratio test.

Based on the scoring patterns of the three latent profile categories, the categories were named and described as follows: Category 1 (Low-level Perceptually Deficient Group, n = 122, 22.85%): This group exhibited consistently low scores across all dimensions of fall risk perception, particularly in fall susceptibility perception (V1) and fall severity perception (V5), reflecting a general lack of awareness regarding mobility limitations and fall risk. Category 2 (Medium-level Condition-dependent Group, n = 263, 49.25%): This group demonstrated moderate levels of fall risk perception overall. Participants exhibited some awareness of environmental factors (V4) and physiological conditions (V2), but showed relatively weak perceptions of personal activity-related risks (V3) and fall severity (V5), relying primarily on external cues rather than proactive risk appraisal. Category 3 (High-level Self-awareness Group, n = 149, 27.90%): This group demonstrated high scores across all dimensions, particularly in perceptions of physiological conditions (V2) and activity-related risks (V3), indicating a high level of self-awareness and vigilance regarding fall risk.

### Univariate Analysis of Fall Risk Perception Profiles Among Cancer Survivors

The univariate analysis presented in Table 3 identified three distinct fall risk perception profiles among cancer survivors undergoing radiotherapy/chemotherapy. Significant differences were observed among the three latent profile groups in chronic disease self-efficacy, duration of illness, diabetes mellitus, fall history, use of walking aids, self-rated health status, caregiver support, and visual status (all *P* < 0.001). In addition, significant differences were found in health literacy, social support, marital status, educational level, tumor stage, hypertension, fear of falling, and sleep status (all *P* < 0.05).

**Table 3:** Univariate analysis of fall risk perception across the three latent profiles (N = 534).

Variable	Group	N (%)	Low level (n=122)	Medium level (n=263)	High level (n=149)	*X^2^*/*F*	*p*
**Age (years)**	<45	149 (27.9%)	32	72	45	0.951	0.917
	45-60	197 (36.9%)	48	95	54		
	>60	188 (35.2%)	42	96	50		
**Sex**	Male	246 (46.1%)	53	122	71	0.500	0.779
	Female	288 (53.9%)	69	141	78		
**Employment status**	Unemployed	75 (14%)	13	43	19	6.671	0.352
	Employed	194 (36.4%)	42	96	56		
	On leave	47 (8.8%)	16	17	14		
	Retired	218 (40.8%)	51	107	60		
**Duration of illness (years)**	<3	264 (49.4%)	88	99	77	52.200	0.000**
	3-5	196 (36.7%)	14	125	57		
	>5	74 (13.9%)	20	39	15		
**Marital status**	Single	90 (16.9%)	30	41	19	13.441	0.009**
	Married	248 (46.4%)	61	123	64		
	Divorced/Widowed	196 (36.7%)	31	99	66		
**Monthly income (RMB)**	<5000	225 (42.1%)	51	108	66	3.002	0.557
	5000-10000	196(36.7%)	50	98	48		
	>10000	113 (21.2%)	21	57	35		
**Treatment modality**	Chemotherapy	98 (18.4%)	24	46	28	4.036	0.401
	Radiotherapy	318 (59.6%)	64	164	90		
	Both	118 (22.0%)	34	53	31		
**Residence**	Rural	167 (31.3%)	40	82	45	2.725	0.605
	Town	151 (28.3%)	37	67	47		
	Urban	216 (40.4%)	45	114	57		
**Education level**	High school	133 (24.9%)	44	51	38	14.334	0.026*
	College	165 (30.9%)	33	90	42		
	Bachelor’s	175 (32.8%)	31	94	50		
	Graduate	61 (11.4%)	14	28	19		
**Tumor stage**	Ⅰ	169 (31.6%)	32	81	56	13.425	0.037*
	Ⅱ	204 (38.2%)	58	95	51		
	Ⅲ	86 (16.1%)	11	51	24		
	Ⅳ	75 (14.1%)	21	36	18		
**Alcohol use**	Yes	197 (36.9%)	44	101	52	0.548	0.760
	No	337 (63.1%)	78	162	97		
**Smoking history**	Yes	143 (26.8%)	30	72	41	0.387	0.824
	No	391 (73.2%)	92	191	108		
**Hypertension**	Yes	341 (63.9%)	64	179	98	9.118	0.010*
	No	193 (36.1%)	58	84	51		
**Diabetes**	Yes	133 (24.9%)	17	84	32	15.745	0.000**
	No	401 (75.1%)	105	179	117		
**Fall history**	Yes	142 (26.6%)	17	89	36	17.543	0.000**
	No	392 (73.4%)	105	174	113		
**Walker**	Use	139 (26%)	10	97	32	37.842	0.000**
	Nonuse	395 (74%)	112	166	117		
**Self assessment of health status**	Bad	100 (18.7%)	9	74	17	35.216	0.000**
	Commonly	238 (44.6%)	71	97	70		
	Good	196 (36.7%)	42	92	62		
**Fear of falling**	Yes	347 (65%)	71	185	91	6.789	0.034*
	No	187 (35%)	51	78	58		
**Exercise**	Hobby	256 (47.9%)	54	125	77	1.513	0.469
	Not hobby	278 (52.1%)	68	138	72		
**Caregiver**	Family	253 (47.4%)	77	98	78	34.520	0.000**
	Nursing workers	158 (29.6%)	19	106	33		
	No caregiver	123 (23%)	26	59	38		
**Vision**	Normal vision	239 (44.8%)	60	133	46	16.178	0.000**
	Vision disorder	295 (55.2%)	62	130	103		
**Sleep**	Insomnia	186 (34.8%)	30	98	58	13.562	0.035*
	Difficulty falling asleep	145 (27.2%)	42	59	44		
	Help with sleep	120 (22.5%)	26	66	28		
	Sleep well	83 (15.5%)	24	40	19		
**Self efficacy**			6.994±2.104	8.411±1.415	7.379±1.856	34.503	0.000**
**Health literacy**			101.92±17.369	106.92±15.389	103.62±16.633	4.567	0.011*
**Social support**			25.79±7.019	27.52±6.972	27.84±6.755	3.458	0.032*

Note: ** indicates *P* < 0.01; * indicates *P* < 0.05.

### Multivariate analysis of latent profiles of fall risk perception

First, multicollinearity was assessed using the VIF and tolerance criteria (VIF < 5; tolerance > 0.20). The results presented in Table 5 show that all independent variables had VIF values < 5 and tolerance values > 0.20, indicating the absence of significant multicollinearity in the model. Multivariate logistic regression analysis was then performed to identify factors associated with fall risk perception among cancer survivors. The three latent profiles of fall risk perception were used as the dependent variable, and factors found to be statistically significant in the univariate analysis were included as independent variables in the regression model (variable assignments are presented in Table 4). The Low-level Perceptually Deficient Group was used as the reference group to explore factors associated with fall risk perception across the latent profiles. The detailed results are presented in Table 5.

**Table 4:** Variable assignment methods.

Independent Variable	Assignment Method
**Duration of illness (years)**	<3（Z_1_ = 0，Z_2_ = 0）, 3–5（Z_1_ = 1，Z_2_ = 0）, > 5（Z_1_ = 0，Z_2_ = 1）
**Marital status**	Single（Z_1_ = 0, Z_2_ = 0）,Married（Z_1_ = 1, Z_2_ = 0）, Divorced/Widowed（Z_1_ = 0, Z_2_ = 1）
**Education level**	High school（Z_1_ = 0, Z_2_ = 0, Z_3_ = 0）, College（Z_1_ = 1, Z_2_ = 0, Z_3_ = 0）, Bachelor’s（Z_1_ = 0, Z_2_ = 1, Z_3_ = 0）, Graduate（Z_1_ = 0, Z_2_ = 0, Z_3_ = 1）
**Tumor stage**	Ⅰ（Z_1_ = 0, Z_2_ = 0, Z_3_ = 0）, Ⅱ（Z_1_ = 1, Z_2_ = 0, Z_3_ = 0）, Ⅲ（Z_1_ = 0, Z_2_ = 1, Z_3_ = 0）, Ⅳ（Z_1_ = 0, Z_2_ = 0, Z_3_ = 1）
**Hypertension**	Yes = 1, No = 0
**Diabetes**	Yes = 1, No = 0
**Fall history**	Yes = 1, No = 0
**Walker**	Use = 1, Nonuse = 0
**Self assessment of health status**	Good（Z_1_ = 0, Z_2_ = 0）, Commonly（Z_1_ = 1, Z_2_ = 0）, Bad（Z_1_ = 0, Z_2_ = 1）
**Fear of falling**	Yes = 1, No = 0
**Caregiver**	Family（Z_1_ = 0, Z_2_ = 0）, Nursing workers（Z_1_ = 1, Z_2_ = 0）, No caregiver（Z_1_ = 0, Z_2_ = 1）
**Vision**	Normal vision = 1, Vision disorder = 0
**Sleep**	Sleep well（Z_1_ = 0, Z_2_ = 0, Z_3_ = 0）, Help with sleep（Z_1_ = 1, Z_2_ = 0, Z_3_ = 0）, Difficulty falling asleep（Z_1_ = 0, Z_2_ = 1, Z_3_ = 0）, Insomnia（Z_1_ = 0, Z_2_ = 0, Z_3_ = 1）
**Self efficacy**	Raw score input
**Health literacy**	Raw score input
**Social support**	Raw score input

**Table 5:** Multivariate analysis of latent profiles of fall risk perception.

Class 1 vs Class 2								Class 1 vs Class 3							Collinearity diagnostics	
Variable	*β*	*SE*	*Wald*	*P*	*OR*	*95%CI*		*β*	*SE*	*Wald*	*P*	*OR*	*95%CI*			
						Low	High						Low	High	Tolerance values	VIF
**Duration of illness (years)**																
**3-5**	2.138	0.389	30.137	0.000**	8.481	3.953	18.195	1.673	0.391	18.301	0.000**	5.326	2.475	11.460	0.818	1.222
**>5**	0.539	0.419	1.650	0.199	1.714	0.753	3.898	−0.110	0.435	0.064	0.800	0.896	0.382	2.101	0.849	1.178
**Marital status**																
**Married**	0.460	0.407	1.278	0.258	1.583	0.714	3.513	0.491	0.407	1.459	0.227	1.635	0.736	3.628	0.459	2.181
**Divorced/Widowed**	0.984	0.439	5.028	0.025*	2.676	1.132	6.325	1.211	0.436	7.703	0.006**	3.357	1.427	7.894	0.457	2.187
**Education level**																
**College**	1.443	0.408	12.513	0.000**	4.233	1.903	9.416	0.928	0.404	5.269	0.022*	2.529	1.145	5.587	0.587	1.703
**Bachelor’s**	1.490	0.398	14.022	0.000**	4.437	2.034	9.678	1.188	0.390	9.282	0.002**	3.280	1.528	7.044	0.595	1.682
**Graduate**	1.271	0.517	6.046	0.014*	3.564	1.294	9.814	1.085	0.508	4.563	0.033*	2.959	1.094	8.008	0.715	1.399
**Tumor stage**																
**Ⅱ**	−0.488	0.365	1.789	0.181	0.614	0.300	1.255	−0.751	0.366	4.217	0.040*	0.472	0.230	0.966	0.652	1.535
**Ⅲ**	0.397	0.505	0.618	0.432	1.488	0.553	4.003	0.232	0.501	0.214	0.644	1.261	0.472	3.367	0.723	1.384
**Ⅳ**	−0.454	0.479	0.896	0.344	0.635	0.248	1.625	−0.835	0.482	3.001	0.083	0.434	0.169	1.116	0.717	1.395
**Hypertension**	1.011	0.307	10.840	0.001**	2.748	1.505	5.016	0.772	0.304	6.455	0.011*	2.165	1.193	3.928	0.929	1.076
**Diabetes**	0.665	0.382	3.026	0.082	1.944	0.919	4.110	0.488	0.389	1.573	0.210	1.629	0.760	3.490	0.874	1.144
**Fall history**	1.122	0.405	7.677	0.006**	3.072	1.389	6.796	0.741	0.411	3.251	0.071	2.099	0.937	4.700	0.846	1.182
**Walker**	1.218	0.438	7.752	0.005**	3.381	1.434	7.970	0.801	0.452	3.134	0.077	2.227	0.918	5.406	0.814	1.228
**Self assessment of health status**																
**Bad**	−0.421	0.520	0.655	0.418	0.657	0.237	1.818	0.048	0.554	0.007	0.932	1.049	0.354	3.105	0.447	2.236
**Commonly**	−1.980	0.497	15.867	0.000*	0.138	0.052	0.366	−0.808	0.528	2.342	0.126	0.446	0.158	1.255	0.499	2.005
**Caregiver**																
**Nursing workers**	1.373	0.380	13.048	0.000*	3.946	1.874	8.311	0.225	0.394	0.327	0.567	1.253	0.579	2.713	0.802	1.246
**No caregiver**	0.289	0.365	0.625	0.429	1.334	0.653	2.729	0.023	0.358	0.004	0.948	1.023	0.508	2.063	0.818	1.222
**Normal vision**	−0.062	0.298	0.043	0.836	0.940	0.524	1.686	−0.957	0.301	10.104	0.001**	0.384	0.213	0.693	0.957	1.045
**Health literacy**	0.021	0.009	5.131	0.024*	1.021	1.003	1.039	0.008	0.009	0.771	0.380	1.008	0.991	1.025	0.886	1.129
**Social support**	0.023	0.021	1.131	0.288	1.023	0.981	1.066	0.044	0.021	4.189	0.041*	1.045	1.002	1.090	0.924	1.082
**Self efficacy**	0.391	0.086	20.931	0.000*	1.479	1.251	1.749	0.018	0.076	0.059	0.808	1.019	0.878	1.182	0.888	1.127

Note: ** indicates *P* < 0.01; * indicates *P* < 0.05; SE, standard error; OR, odds ratio; CI, confidence interval; VIF, variance inflation factor. Class 1 = Low-level Perceptually Deficient Group; Class 2 = Medium-level Condition-dependent Group; Class 3 = High-level Self-awareness Group.

## Discussion

This study innovatively integrated LPA with the HBM, overcoming the limitations of traditional variable-centered analyses in assessing cancer survivors’ fall risk perception [31]. The results indicated that the overall fall risk perception score among cancer survivors was 89.337 ± 31.071, representing a moderately high level. Using LPA, three distinct subgroups were identified based on fall risk perception: the Low-level Perceptually Deficient Group, the Medium-level Condition-dependent Group, and the High-level Self-awareness Group. Furthermore, multivariate logistic regression analysis revealed that membership in the Low-level Perceptually Deficient Group was associated with lower educational attainment, hypertension, a history of falls, use of walking aids, and lower levels of health literacy, self-efficacy, and social support. These findings provide a valuable basis for developing targeted risk management strategies tailored to different subgroups.

Our findings revealed that cancer survivors with a disease duration of 3–5 years were more likely to exhibit moderate-to-high levels of fall risk perception, whereas those with stage II tumors were more likely to belong to the High-level Self-awareness Group (OR = 0.472). This nonlinear relationship may reflect dynamic psychological adaptation during cancer progression, suggesting that the middle stage of the disease trajectory may represent a critical period for the development of fall risk perception. This finding is consistent with the results reported by Zhao J et al. [39]. The HBM provides further insight into these findings by proposing that individuals must first recognize their susceptibility to falls and the potential severity of fall-related consequences before adopting preventive behaviors [27]. Patients with a disease duration of less than 3 years may focus primarily on survival-related concerns and therefore regard symptoms such as fatigue and dizziness as unavoidable side effects of treatment. In contrast, patients with a disease duration exceeding 5 years may develop perceptual biases because of psychological adaptation or diminished attention to risk. The HBM further posits that the adoption of health-related behaviors results from weighing the perceived benefits and barriers of preventive actions, while self-efficacy facilitates the translation of behavioral intentions into action [28]. Patients with a disease duration of 3–5 years may enter a relatively stable phase of disease management, shifting toward proactive life management and greater internalization of health information, thereby enhancing their perception of fall risk. Previous studies have shown that HBM-based education, progressive training, and environmental support can improve risk awareness and reduce fall incidence by 23% [40]. Tian Lu et al. [41] demonstrated that patients at home during treatment intervals remain at elevated risk of falls, highlighting the need to extend fall prevention efforts beyond the hospital setting. Therefore, we recommend establishing transitional fall prevention clinics to facilitate the integration of preventive resources across healthcare settings. Health education interventions should target all cancer survivors, particularly those in the early and advanced stages of disease, by emphasizing susceptibility to falls and the severity of potential consequences while enhancing self-efficacy to promote sustained health-protective behaviors [42].

This study found that cancer survivors with higher levels of social support tended to have greater fall risk perception. This association may be attributed to the empowering effect of social support, which enables patients to identify and address potential risks more proactively, thereby fostering a state of active vigilance. According to the stress-buffering model [43], social support enhances individuals’ confidence in managing health threats, strengthens perceived susceptibility and perceived severity, and promotes the adoption of preventive behaviors. Poh FJX et al. [44] further identified psychological support, family involvement, and self-management as key mediators of this relationship. Notably, divorced or widowed survivors were more likely to exhibit moderate-to-high levels of fall risk perception than their married counterparts, reflecting a dual challenge [45]. On the one hand, the lack of instrumental support may compel greater self-reliance, thereby reinforcing cautiousness. On the other hand, emotional deprivation may intensify feelings of loneliness and catastrophic thinking, collectively increasing awareness of fall-related risks. Within fall prevention systems, professional caregivers (e.g., nursing assistants) contribute expertise in risk assessment and intervention, whereas informal caregivers (e.g., family members) provide emotional support and assistance with daily living activities. High-quality caregiving not only offers physical protection but also promotes self-management awareness. Previous studies have confirmed that HBM-based nurse–patient interactions improve patients’ confidence and engagement in preventive behaviors [46]. Therefore, we recommend systematically evaluating patients’ marital status and social support networks to identify support gaps and optimize care strategies [44]. Guided by the HBM framework, personalized interventions should integrate risk awareness, skills training, and resource linkage to enhance both fall risk perception and adherence to self-management behaviors [46].

Our study found that cancer survivors with higher educational attainment were more likely to exhibit moderate-to-high levels of fall risk perception, demonstrating a gradient relationship. This pattern may reflect the “double-edged sword” effect of health literacy described by Kuhlenschmidt ML et al. [47]. Patients with higher levels of health literacy and self-efficacy tend to maintain an appropriate level of risk awareness. Evidence suggests that health literacy mediates the relationship between social factors and health outcomes [48]. Individuals with limited health literacy often experience difficulty accurately evaluating their functional status, including balance, visual function, and medication-related adverse effects, which may hinder the effective identification of fall risks [49]. These information-processing barriers can impair self-management and increase the risk of falls. Self-efficacy plays a pivotal role in this process [50]. It enables patients to translate risk perception into preventive behaviors, such as seeking health information or engaging in balance training exercises. The belief that they can effectively manage potential challenges allows patients to acknowledge risks rationally, thereby fostering proactive vigilance and sustaining moderate-to-high levels of risk awareness. Hou Chunhua further confirmed a positive association between educational level and fall risk awareness [51]. Therefore, for individuals with low educational attainment, limited health literacy, and low self-efficacy, we recommend implementing accessible educational interventions supported by visual materials and involving family members and caregivers. A four-component strategy integrating cognition, behavior, environment, and social support, implemented through adaptive communication, structured skills training, psychological empowerment, and multidisciplinary collaboration, may help interrupt the cycle of risk and achieve effective fall prevention [52].

Our study found that cancer survivors with comorbid hypertension were more likely to underestimate their fall risk, whereas those reporting a “fair” self-rated health status generally exhibited a moderate level of fall risk perception, consistent with the findings of Tian Lu et al. [41]. Comorbidities and self-rated health status appear to be important factors influencing fall risk perception. Hypertension increases cardiovascular burden and may impair functional status, thereby affecting the accurate perception of fall risk [53]. Frailty among older patients with hypertension may interact with cancer-related functional decline, contributing to a “cancer–frailty–fall” cycle [54]. In addition, antihypertensive medications may induce orthostatic hypotension and dizziness, significantly increasing the risk of falls among cancer survivors with impaired physiological regulatory capacity. However, these objective risk factors may not be adequately translated into subjective risk awareness, highlighting the mediating role of health beliefs. Research has confirmed that health beliefs mediate the relationship between knowledge and health-related behaviors among patients with hypertension [55]. Even when adequate knowledge is present, individuals may be unlikely to adopt preventive behaviors without perceiving their susceptibility to risk and the benefits of preventive actions. For example, adherence to functional exercise among patients receiving radiotherapy for head and neck cancer has been shown to be positively associated with health belief levels [56]. Strengthening the belief that exercise is beneficial is therefore essential for promoting participation in rehabilitation. We recommend adopting integrated intervention strategies. First, assessments of comorbidities, polypharmacy, and frailty should be incorporated into routine fall risk screening to identify patients with high objective risk but low subjective risk awareness. Second, health beliefs should be strengthened through personalized interventions, such as motivational interviewing [57]. Finally, interdisciplinary collaboration should integrate the HBM and structured exercise prescriptions into cancer rehabilitation pathways [58]. This approach represents a conceptual transition from disease-centered care to patient-centered care and may facilitate sustainable fall prevention through the combined enhancement of health beliefs and physical function.

Our findings indicate that cancer survivors with a history of falls or those using walking aids were more likely to exhibit a moderate level of fall risk perception. Previous studies have shown that cancer survivors undergoing radiotherapy and chemotherapy frequently experience geriatric syndromes, including sarcopenia, osteoporosis, and physical frailty [59]. Among these patients, 57.7% experience reductions in muscle mass and strength, resulting in impaired balance and sensory function. In addition, treatment-induced tissue fibrosis and joint stiffness may further restrict mobility, contributing to a frailty-related condition that increases the risk of falls. Notably, fall history showed only marginal statistical significance in the High-level Self-awareness Group (*P* = 0.077), suggesting that elevated levels of fall risk perception may be influenced by more complex psychosocial factors. From the perspective of the HBM, a previous fall may serve as a powerful cue to action, reducing the cognitive bias that “it will not happen to me.” Conversely, the use of walking aids may be associated with perceived stigma, as these devices can be viewed as symbols of frailty. This perception may increase psychological burden and hinder accurate assessment of fall risk. However, clinical guidelines indicate that, for cancer survivors with muscle weakness or balance impairment, walking aids provide physical support, enhance confidence in walking, and reduce fear of falling [60]. To strengthen fall prevention efforts, we recommend establishing an integrated system that incorporates preventive education, assistive device adaptation, multidisciplinary collaboration, and intelligent technology applications [61,62]. Such an approach would facilitate a transition from passive responses to proactive prevention. Supervised elastic-band exercise programs should be performed two to three times per week for 30 minutes per session, beginning at 30%–40% of one-repetition maximum (1RM) and progressing to 50%–60% of 1RM [63,64], to improve lower-extremity strength and balance. Future research should focus on developing cancer-specific fall risk prediction models and validating intelligent monitoring technologies in clinical practice [61,62].

### Limitation

This study has several limitations. First, the cross-sectional study design precludes the inference of causal relationships among variables. The assessment of key constructs, including fall risk perception and self-efficacy, relied exclusively on patient-reported outcome measures, which may be subject to recall and social desirability biases. Second, the sample was recruited from a single hospital, which may introduce selection bias and limit the generalizability of the findings. However, the hospital selected for this study is a provincial regional medical center in Shandong Province. It operates three campuses with approximately 4,000 licensed beds, including 400 beds in the oncology department, 80 beds in the hematology department, and 35 beds in the radiotherapy department. Its healthcare services extend to multiple surrounding cities and serve a population of approximately 8 million people. The hospital admits more than 20,000 patients with cancer annually and is therefore representative of regional oncology care. In addition, potential confounding factors were not fully controlled. Future research should employ longitudinal study designs, expand the sample size, and implement more rigorous control of confounding variables to validate the present findings. From a clinical application perspective, although this study identified high-risk subgroups, the practical implementation of these findings depends on the development of a feasible risk stratification tool. The current model relies on a relatively complex set of variables that may require simplification to facilitate efficient screening in busy clinical settings. Furthermore, this study did not perform stratified analyses according to cancer type, which may have obscured differences associated with variations in cancer subtypes. In addition, the sample sizes for certain cancer types were insufficient to support reliable subgroup analyses. Future studies should recruit larger samples to investigate the potential moderating effects of psychosocial factors across different cancer types.

## Conclusion

This study employed LPA to classify cancer survivors undergoing radiotherapy/chemotherapy into three distinct latent classes based on their levels of fall risk perception: the High-level Self-awareness Group, the Medium-level Condition-dependent Group, and the Low-level Perceptually Deficient Group. These findings provide a theoretical foundation for the development of tailored intervention strategies. Significant differences were observed among the three latent classes in key factors, including self-efficacy, health literacy, and social support. Notably, the Low-level Perceptually Deficient Group was identified as the priority population for intervention.

A stratified management approach is recommended in clinical practice. For the High-level Self-awareness Group, interventions should focus on maintaining and reinforcing existing support systems. For the Medium-level Condition-dependent Group, targeted training should be implemented to address gaps in risk perception and preventive behaviors. For the Low-level Perceptually Deficient Group, a family-centered collaborative support system should be established and supplemented with psychological interventions, such as cognitive behavioral therapy. In addition, attention should be given to the moderating effects of influential factors, such as educational attainment, across different pathways. Future research should focus on the following: (1) validating the applicability of the classification criteria proposed in this study across diverse populations and investigating the interaction between cancer type and fall risk perception, with further validation through multicenter studies; (2) developing an artificial intelligence-based automated classification tool to improve the efficiency of clinical screening; and (3) conducting randomized controlled trials of subgroup-specific intervention programs to systematically evaluate their effects on patients’ quality of life and treatment outcomes, thereby promoting the refinement and systematization of fall prevention strategies.

### Declaration of generative AI and AI-assisted technologies in the writing process

No AI tools/services were used during the preparation of this work.

## Supporting information

S1 Fig Health Belief Model-Theoretical Framework. (PDF)

S2 Fig Distribution of characteristics in three potential categories of Perception of fall risk. (PDF)

S1 File Ethics Approval Certificate. (PDF)
