# Supplementary material for: Perception of fall risk among cancer survivors receiving Radiotherapy/Chemotherapy in China: A latent profile analysis
Source: PLoS One. 2026 Sep 24;21(9):e0358845. doi: 10.1371/journal.pone.0358845 (PMC13606947; doi:10.1371/journal.pone.0358845)
Supplement: Supplementary file 3 [file pone.0358845.s003.pdf]

Ethics Approval Certificate
Medical Ethics Expert Committee, Zibo Central Hospital

Approval Number: 2025 Research No. 181

Item	Details
**Project Title**	Latent Profile Analysis of Fall Risk Perception in Cancer Patients
**Project Source**	Zibo Central Hospital
**Research Unit**	Zibo Central Hospital
**Principal Investigator / Department**	Jing Xuebing / Drug Clinical Trial Office
**Review Method**	□ Full Board Review ☑ Expedited Review
**Review Date**	May 28, 2025
**Review Location**	/
**Review Category**	☑ Initial Review<br>□ Amendment Review<br>□ Re-review<br>□ Protocol Violation Review<br>□ Annual/Continuing Review<br>□ SAE/Unexpected Event Review<br>□ Study Suspension/Termination Review<br>□ Final Report Review
**Reviewed Documents**	1. Application Form for Clinical Research Ethics Review<br>2. Study Protocol<br>3. Informed Consent Form
**Review Committee Members**	Duan Bide, Fang Xiaojie
**Research Type**	1. □ Interventional (Experimental Research)<br>□ Drug □ Device □ Medical Technology □ Other<br>2. ☑ Observational Research<br>□ Survey ☑ Data Collection □ Genetic Research □ Epidemiology □ Other<br>3. □ Animal Experiment
**Review Decision**	1. Decision:<br>☑ Approved

Item	Details
	□ Approved with Modifications<br>□ Re-review Required<br>□ Continue Research<br>□ Not Approved<br>□ Suspend or Terminate Research<br>2. Review Frequency:<br>□ 3 months □ 6 months □ 12 months<br>3. Approval Validity Period: 1 year

Review Comments:

After review by our Medical Ethics Committee, the investigator’s qualifications and experience meet the study requirements; the study protocol complies with scientific and ethical principles; the informed consent process and content are appropriate; the risks to participants are 可控 (controllable), and the risk level is appropriate relative to the anticipated benefits. The applicant has committed to adhering to ethical guidelines and accepting ongoing supervision and inspection by the committee. The study is approved to proceed.

Notes:

①This study must be initiated within one year of the ethics approval date; otherwise, this approval becomes invalid.

② The study must be conducted in accordance with ethical principles and the approved protocol to protect participants’ rights.

③Any changes in principal investigator, study protocol, or informed consent form require a amendment review submission.

④A progress report must be submitted one month before the scheduled continuing review deadline according to the specified frequency.

⑤Any protocol violations must be reported promptly.

⑥If the study is suspended or terminated early, a report must be submitted promptly.

⑦A final report must be submitted upon study completion.

Signature of Chair/Vice Chair:

Date: May 28, 2025

Medical Ethics Expert Committee

Zibo Central Hospital

Address: No. 10 Nanhai Road, Zhangdian District, Zibo City, Shandong Province, China, 255036

Telephone: 0533-2360221
