# Supplementary material for: Perception of fall risk among cancer survivors receiving Radiotherapy/Chemotherapy in China: A latent profile analysis
Source: PLoS One. 2026 Sep 24;21(9):e0358845. doi: 10.1371/journal.pone.0358845 (PMC13606947; doi:10.1371/journal.pone.0358845)
Supplement: Supplementary file 4 [file pone.0358845.s005.pdf]

Response to reviewers

Dear reviewers：

Thank you very much for reading this article in your busy schedule, and thank the editorial team and reviewers for their feedback on our manuscript, and provide many constructive suggestions for revision, which have greatly helped us to further improve the article. We have carefully considered and analyzed the opinions of peer reviewers, and on this basis, we have revised the articles one by one.

To reviewer' comments

MAJOR COMMENTS

#1： Absence of Cancer-Type Information.One of the most significant limitations is the lack of information regarding cancer diagnoses.Different cancer types have substantially different fall risk profiles due to variations in:• Disease burden;• Functional impairment;• Neurotoxicity;• Treatment side effects.Variables such as breast cancer, lung cancer, gastrointestinal cancer, hematological malignancies, and metastatic disease should be reported and considered in the analysis.

Author response： We thank the reviewer for the valuable comments. We fully agree that cancer type is a key variable influencing fall risk perception, as different cancer types vary substantially in disease burden, functional impairment, neurotoxicity, and treatment-related side effects. The initial design of this study focused on psychosocial factors and did not prespecify cancer type as a core variable. Moreover, the sample sizes for certain cancer types were too small to support stable subgroup analyses. In response, we have added a "Type of tumor" row in Table 1, reporting the counts and percentages of each cancer type. We explicitly acknowledge that cancer type information was not systematically analyzed as a limitation, which has been stated in the Limitations section. Additionally, in the Conclusion section, we recommend that future studies include cancer type as a key covariate and explore the interaction mechanisms between cancer type and fall risk perception.(P8-9 L255; P22 L462-466; P23 L485-486).

#2： Single-Center Convenience Sampling.Participants were recruited from a single tertiary hospital.This introduces potential:•Selection bias•Regional bias•Institutional practice bias.which limits external validity and generalizability.

Author response： We thank the reviewer for the rigorous correction. We fully agree that single-center convenience sampling carries risks of selection bias, regional bias, and institutional practice bias, which limit the generalizability and external validity of the findings. However, the hospital selected for this study is a provincial-level regional medical center in Shandong Province. It currently operates three campuses with approximately 4,000 open beds, including 400 beds in the oncology department, 80 beds in the hematology department, and 35 beds in the radiotherapy department. Its medical services cover multiple surrounding cities, serving a population of approximately 8 million. The hospital admits more than 20,000 cancer patients annually, making it representative in the field of regional oncology diagnosis and treatment. We have revised the Limitations and Conclusion sections accordingly, and all changes are highlighted in red. Thank you again for your valuable comment.(P22 L450-456).

#3： Incomplete Reporting of Missing Data. The manuscript states that multiple imputation was performed; however, critical information is missing:•Percentage of missing data•Missing data mechanism•Number of imputations•Imputation procedures.This information is necessary for reproducibility and transparency.

Author response： We thank the reviewer for the rigorous correction. Regarding the issue of incomplete reporting of missing data, we have carefully verified each item and respond as follows:A total of 560 questionnaires were distributed in this study. Following rigorous quality control, 26 invalid responses were excluded based on the inclusion and exclusion criteria: 1) contradictory answers (n=15): logically inconsistent responses; 2) patterned responses (n=8): overly uniform response patterns; and 3) short completion time (n=3): completion time of less than 3 minutes, far below the average completion time. The final valid sample size was 534 cases, and all study variables had no missing values in the dataset ultimately included for analysis.The statement "Missing data were handled using multiple imputation" in the original manuscript was a writing error and did not reflect the actual data processing procedure. We sincerely apologize for this mistake and have corrected it in the revised manuscript.(P7 L223-225).

#4： Lack of Multicollinearity Assessment.Several psychosocial variables included in the regression model are conceptually correlated, including:•Health literacy•Self-efficacy•Social support.The authors should report:•Variance Inflation Factors (VIF)•Tolerance values•Collinearity diagnostics to demonstrate model stability.

Author response: Thank you for raising this important methodological issue. Your concern regarding potential multicollinearity among psychosocial variables such as health literacy, self-efficacy, and social support is very reasonable. As requested, we conducted collinearity diagnostics for all independent variables included in the regression model. The results showed: health literacy (VIF=1.129, Tolerance=0.886); self-efficacy (VIF=1.127, Tolerance=0.888); social support (VIF=1.082, Tolerance=0.924). All VIF values were < 5 and all Tolerance values were > 0.2. The results have been added to Table 5 (see Table 5 for details).(P8 L242-245; P14 L299-302;P16-17 L310-311).

#5: Limited Reporting of LPA Classification Quality.Although AIC, BIC, entropy, LMRT, and BLRT were reported, additional classification quality indices are recommended: Classification accuracy for each latent class.

Author response: Thank you for this professional and important suggestion. We fully agree that reporting only the overall Entropy is insufficient for comprehensively evaluating the classification quality of LPA, and that reporting classification accuracy for each latent class is essential for verifying the reliability and interpretability of the model. We extracted the average posterior probabilities from the Mplus output file as the core indicator for evaluating classification accuracy for each latent class. The average posterior probabilities for the three latent classes were 1.000, 1.000, and 0.993, respectively, all significantly higher than the recommended threshold of 0.80, indicating that the LPA model in this study has excellent classification accuracy with good distinction among latent classes. The classification accuracy for each latent class has now been added to Table 2, and all revisions have been highlighted in red in the revised manuscript. Thank you again for your rigorous scrutiny of LPA methodological quality.(P8 L236-238; P11 L266-269; P12 L284).

#6: Cross-Sectional Design.The cross-sectional nature of the study precludes causal inference.Terms such as "influencing factors" and "predictors" should be used cautiously and replaced with "associated factors" where appropriate.

Author response: Thank you for pointing out this important issue. We fully agree that the cross-sectional design precludes causal inference, and the use of causally suggestive language could mislead readers. We have systematically reviewed and revised the entire manuscript accordingly, and all changes have been highlighted in red in the revised manuscript.(P1 L20; P2 L42; P4 L106 L112; P5 L148).

Areas for Improvement

#7： Language Quality.Numerous grammatical and stylistic issues are present throughout the manuscript, including:•Inconsistent tense usage•Missing articles•Subject-verb disagreement•Non-native English phrasing.Professional language editing is strongly recommended.

Author response： Thank you for pointing out the language quality issues. We have commissioned a professional team to systematically edit the entire manuscript, correcting problems such as inconsistent tense usage, missing articles, subject-verb disagreement, and non-native English phrasing. All are marked in red.

#8： Data Consistency.There appears to be inconsistency regarding the reported sex distribution of participants between the Results section and Table 1.（line236 Results: “including 246 (51.90%) 236men and 228 (48.10%) women.”；line238：Table 1:Male=46.1%）Female 288 (53.9%)…The authors should carefully verify all reported statistics.

Author response： Thank you very much for pointing out this data inconsistency issue. We immediately verified all raw data and the numbers reported in the manuscript item by item. Upon verification, we confirmed that a data inconsistency did exist. This error has been corrected in the sex distribution data in the Results section. We sincerely apologize for this oversight in data entry and proofreading. We have now rechecked all data in the manuscript and confirmed that no other errors exist. We apologize again and thank you for your rigorous review.(P8 L253).

#9： Overly Lengthy Discussion.The Discussion section could be reduced by approximately 20-30% to improve focus and readability.

Author response： Thank you for your comment. We have streamlined the Discussion section as requested, primarily by removing redundant statements, excessive modifiers, and overly extended content. All revisions have been highlighted in red in the revised manuscript.(P18-19 L325-350; P19 L352-354; P19-21 L373-443).
