# Supplementary material for: Perception of fall risk among cancer survivors receiving Radiotherapy/Chemotherapy in China: A latent profile analysis
Source: PLoS One. 2026 Sep 24;21(9):e0358845. doi: 10.1371/journal.pone.0358845 (PMC13606947; doi:10.1371/journal.pone.0358845)
Supplement: Supplementary file 5 [file pone.0358845.s006.docx]

**Response to reviewers**

Dear reviewers：

Thank you very much for reading this article in your busy schedule, and thank the editorial team and reviewers for their feedback on our manuscript, and provide many constructive suggestions for revision, which have greatly helped us to further improve the article. We have carefully considered and analyzed the opinions of peer reviewers, and on this basis, we have revised the articles one by one.

**To reviewer’ comments**

**#1：**"All of these measures should be headings and if they are APA level 4 then they are wrong but I am not sure what format you are using"

**Author response：**We thank the reviewer for this valuable suggestion. We have revised the headings of all measures in the Methods section. Each scale (General Information Questionnaire, Fall Risk Perception Scale, Social Support Rating Scale, Health Literacy Management Scale, and Self-Efficacy to Manage Chronic Disease Scale) is now presented as an independent Level 3 heading (left-aligned and bold), in accordance with the journal's formatting requirements. The revisions have been highlighted in the revised manuscript.(P6 L175, L186, L196;P7 L207).

**#2：**"You have a general list here. So it is not a sentence or a paragraph. You can make a table, attached the survey as an appendix, or simply say it included X number of questions related to and name a few and then say see Table or Appendix".

**Author response：**We agree with the reviewer that listing all general information items in the text is inappropriate. We have revised this section to a single sentence (General demographic information includes 23 variables, such as gender and age; for detailed information, please refer to the appendix., as suggested. The revision has been highlighted in the manuscript.(P5 L164-166).

**#3：**The Health Literacy Management Scale was originally developed by Jordan et al. [35] and the was adapted into Chinese by Sun Haolin in 2012 [36]. Shuiyuan [34] in 1986, is was used to.

**Author response：**We thank the reviewer for the rigorous correction. Thank you very much for pointing out the grammatical and expressive errors in the scale citation section; we sincerely apologize for these oversights resulting from carelessness. The revised sections have been marked in red.(P6 L187-188,197-200).

**#4：**You need to set up a at analytic section and explicitly name the variables and what analyzes you will do. The M+ analysis area great. But the demographic data goes into the participant section.

**Author response：**Thank you very much for your guidance on the statistical analysis section. It should be noted that we have already clearly reported in the "Statistical Analysis" section the statistical software used in this study (Mplus 8.3 and SPSS 26.0), the analytical methods applied to each variable, the metrics for evaluating the fit of the LPA model, the diagnostic criteria for multicollinearity, and the significance levels. Additionally, the descriptive statistics for demographic data have been presented, as required, in the "Demographic and Clinical Characteristics" subsection at the beginning of the "Results" section (Table 1); they are not repeated in the Statistical Analysis section. General information is provided in the dedicated Participant Section within the Study Design.(P5 L164-166; P8 L245-260).
