# Supplementary material for: Perception of fall risk among cancer survivors receiving Radiotherapy/Chemotherapy in China: A latent profile analysis
Source: PLoS One. 2026 Sep 24;21(9):e0358845. doi: 10.1371/journal.pone.0358845 (PMC13606947; doi:10.1371/journal.pone.0358845)
Supplement: Supplementary file 6 [file pone.0358845.s007.docx]

**Response to reviewers**

Dear reviewers：

Thank you very much for reading this article in your busy schedule, and thank the editorial team and reviewers for their feedback on our manuscript, and provide many constructive suggestions for revision, which have greatly helped us to further improve the article. We have carefully considered and analyzed the opinions of peer reviewers, and on this basis, we have revised the articles one by one.

**To reviewer’ comments**

**#1：**"You added Participants at 164 with a statement of looking towards the table that is still in the results. Participant data goes up into the method---please move it up to that point."

**Author response：**We thank the editor for this valuable suggestion. We have moved the descriptive participant data to the Methods section under the “Participants” subsection (around line 164) , while retaining the reference to the relevant table. (P4 L164).

**#2：**The redundant heading at line 262 should be removed.

**Author response：**We have deleted that heading as requested and checked the surrounding text to ensure no loss of coherence.

**#3：**The heading at line 302 appears as an orphan/widow heading and should be forced to the next page.

**Author response：**We thank the editor for the rigorous correction. We have inserted a hard page break before that heading, ensuring it now appears at the top of the following page, in compliance with the journal’s formatting requirements.(P12 L303).
