# Supplementary material for: Perception of fall risk among cancer survivors receiving Radiotherapy/Chemotherapy in China: A latent profile analysis
Source: PLoS One. 2026 Sep 24;21(9):e0358845. doi: 10.1371/journal.pone.0358845 (PMC13606947; doi:10.1371/journal.pone.0358845)
Supplement: Peer review history

# Peer review: Decision Letter 0

**DOI:** 10.1371/journal.pone.0358845.r001

**Authors:** Mary Diane Clark

7 Jun 2026

Dear Dr. Zhang,

Thank you for submitting your manuscript to PLOS ONE. After careful consideration, we feel that it has merit but does not fully meet PLOS ONE’s publication criteria as it currently stands. Therefore, we invite you to submit a revised version of the manuscript that addresses the points raised during the review process.

Please submit your revised manuscript by Jul 22 2026 11:59PM. If you will need more time than this to complete your revisions, please reply to this message or contact the journal office at plosone@plos.org. When you're ready to submit your revision, log on to https://www.editorialmanager.com/pone/ and select the 'Submissions Needing Revision' folder to locate your manuscript file.

- A letter that responds to each point raised by the academic editor and reviewer(s). You should upload this letter as a separate file labeled 'Response to Reviewers'.
- A marked-up copy of your manuscript that highlights changes made to the original version. You should upload this as a separate file labeled 'Revised Manuscript with Track Changes'.
- An unmarked version of your revised paper without tracked changes. You should upload this as a separate file labeled 'Manuscript'.

If you would like to make changes to your financial disclosure, please include your updated statement in your cover letter. Guidelines for resubmitting your figure files are available below the reviewer comments at the end of this letter.

If applicable, we recommend that you deposit your laboratory protocols in protocols.io to enhance the reproducibility of your results. Protocols.io assigns your protocol its own identifier (DOI) so that it can be cited independently in the future. For instructions see: https://journals.plos.org/plosone/s/submission-guidelines#loc-laboratory-protocols. Additionally, PLOS ONE offers an option for publishing peer-reviewed Lab Protocol articles, which describe protocols hosted on protocols.io. Read more information on sharing protocols at https://plos.org/protocols?utm_medium=editorial-email&utm_source=authorletters&utm_campaign=protocols.

As the corresponding author, your ORCID iD is verified in the submission system and will appear in the published article. PLOS supports the use of ORCID, and we encourage all coauthors to register for an ORCID iD and use it as well. Please encourage your coauthors to verify their ORCID iD within the submission system before final acceptance, as unverified ORCID iDs will not appear in the published article. *Only*  the individual author can complete the verification step; PLOS staff *cannot*  verify ORCID iDs on behalf of authors.

We look forward to receiving your revised manuscript.

Kind regards,

Mary Diane Clark, PhD

Academic Editor

PLOS One

Journal requirements:

When submitting your revision, we need you to address these additional requirements.

1. Please ensure that your manuscript meets PLOS ONE's style requirements, including those for file naming. The PLOS ONE style templates can be found at

https://journals.plos.org/plosone/s/file?id=wjVg/PLOSOne_formatting_sample_main_body.pdf and

https://journals.plos.org/plosone/s/file?id=ba62/PLOSOne_formatting_sample_title_authors_affiliations.pdf

2. Your ethics statement should only appear in the Methods section of your manuscript. If your ethics statement is written in any section besides the Methods, please move it to the Methods section and delete it from any other section. Please ensure that your ethics statement is included in your manuscript, as the ethics statement entered into the online submission form will not be published alongside your manuscript.

3. In the online submission form you indicate that your data is not available for proprietary reasons and have provided a contact point for accessing this data. Please note that your current contact point is a co-author on this manuscript. According to our Data Policy, the contact point must not be an author on the manuscript and must be an institutional contact, ideally not an individual. Please revise your data statement to a non-author institutional point of contact, such as a data access or ethics committee, and send this to us via return email. Please also include contact information for the third party organization, and please include the full citation of where the data can be found.

If the reviewer comments include a recommendation to cite specific previously published works, please review and evaluate these publications to determine whether they are relevant and should be cited. There is no requirement to cite these works unless the editor has indicated otherwise.

Additional Editor Comments:

Thank you for an important article on an important topic. The reviewer notes some weaknesses in you reporting of the method and statistics. In addition, you have gone beyond the data a bit in your discussion. Please address these issues, especially those as listed by the reviewer.

[Note: HTML markup is below. Please do not edit.]

Reviewers' comments:

Reviewer's Responses to Questions

**Comments to the Author**

1. Is the manuscript technically sound, and do the data support the conclusions?

Reviewer #1: Partly

**********

2. Has the statistical analysis been performed appropriately and rigorously?

Reviewer #1: Yes

**********

3. Have the authors made all data underlying the findings in their manuscript fully available?

The PLOS Data policy

Reviewer #1: No

**********

4. Is the manuscript presented in an intelligible fashion and written in standard English?

Reviewer #1: Yes

**********

Reviewer #1: This study addresses an important and clinically relevant topic in oncology nursing, namely the heterogeneity of fall risk perception among cancer survivors undergoing radiotherapy and/or chemotherapy. The application of Latent Profile Analysis (LPA) is appropriate and provides additional insights beyond traditional variable-centered approaches. The sample size is adequate, and the findings may have practical implications for the development of stratified fall-prevention interventions.

However, after a detailed evaluation, I believe the manuscript requires substantial revision before it can be considered for publication.

MAJOR COMMENTS

1. Absence of Cancer-Type Information

One of the most significant limitations is the lack of information regarding cancer diagnoses.

Different cancer types have substantially different fall risk profiles due to variations in:

• Disease burden

• Functional impairment

• Neurotoxicity

• Treatment side effects

Variables such as breast cancer, lung cancer, gastrointestinal cancer, hematological malignancies, and metastatic disease should be reported and considered in the analysis.

2. Single-Center Convenience Sampling

Participants were recruited from a single tertiary hospital.

This introduces potential:

• Selection bias

• Regional bias

• Institutional practice bias

which limits external validity and generalizability.

3. Incomplete Reporting of Missing Data

The manuscript states that multiple imputation was performed; however, critical information is missing:

• Percentage of missing data

• Missing data mechanism

• Number of imputations

• Imputation procedures

This information is necessary for reproducibility and transparency.

4. Lack of Multicollinearity Assessment

Several psychosocial variables included in the regression model are conceptually correlated, including:

• Health literacy

• Self-efficacy

• Social support

The authors should report:

• Variance Inflation Factors (VIF)

• Tolerance values

• Collinearity diagnostics

to demonstrate model stability.

5. Limited Reporting of LPA Classification Quality

Although AIC, BIC, entropy, LMRT, and BLRT were reported, additional classification quality indices are recommended: Classification accuracy for each latent class.

6. Cross-Sectional Design

The cross-sectional nature of the study precludes causal inference.

Terms such as “influencing factors” and “predictors” should be used cautiously and replaced with “associated factors” where appropriate.

Areas for Improvement

Language Quality

Numerous grammatical and stylistic issues are present throughout the manuscript, including:

• Inconsistent tense usage

• Missing articles

• Subject-verb disagreement

• Non-native English phrasing

Professional language editing is strongly recommended.

Data Consistency

There appears to be inconsistency regarding the reported sex distribution of participants between the Results section and Table 1.（line236 Results:

“including 246 (51.90%) 236men and 228 (48.10%) women. ” ；line238：Table 1:Male = 46.1%）Female 288 (53.9%)…

The authors should carefully verify all reported statistics.

Excessive Speculation

Several interpretations in the Discussion extend beyond the available data and should be presented more cautiously.

Overly Lengthy Discussion

The Discussion section could be reduced by approximately 20–30% to improve focus and readability.

Major Strengths

• Clinically important topic in cancer survivorship care.

• Adequate sample size for latent profile modeling.

• Appropriate use of Latent Profile Analysis.

• Theoretical support from the Health Belief Model.

Major Weaknesses

• Lack of cancer-type stratification.

• Single-center design.

• Insufficient reporting of missing data handling.

• Absence of multicollinearity diagnostics.

• Limited reporting of latent profile classification accuracy.

7. Recommendation

Decision: Major Revision

Rationale

The manuscript addresses an important clinical issue and demonstrates potential scientific value. However, substantial revisions are required to improve methodological transparency, strengthen statistical reporting, address key confounding variables, and ensure compliance with journal policies.

**********

what does this mean?). If published, this will include your full peer review and any attached files.

If you choose “no”, your identity will remain anonymous but your review may still be made public.

**Do you want your identity to be public for this peer review?**  For information about this choice, including consent withdrawal, please see our Privacy Policy

Reviewer #1: No

**********

[NOTE: If reviewer comments were submitted as an attachment file, they will be attached to this email and accessible via the submission site. Please log into your account, locate the manuscript record, and check for the action link "View Attachments". If this link does not appear, there are no attachment files.]

To ensure your figures meet our technical requirements, please review our figure guidelines: https://journals.plos.org/plosone/s/figures

You may also use PLOS’s free figure tool, NAAS, to help you prepare publication quality figures: https://journals.plos.org/plosone/s/figures#loc-tools-for-figure-preparation.

NAAS will assess whether your figures meet our technical requirements by comparing each figure against our figure specifications.

# Author comment: Author response to Decision Letter 1

**DOI:** 10.1371/journal.pone.0358845.r002

2 Jul 2026

Response to reviewers

Dear reviewers：

Thank you very much for reading this article in your busy schedule, and thank the editorial team and reviewers for their feedback on our manuscript, and provide many constructive suggestions for revision, which have greatly helped us to further improve the article. We have carefully considered and analyzed the opinions of peer reviewers, and on this basis, we have revised the articles one by one.

To reviewer’ comments

MAJOR COMMENTS

#1：Absence of Cancer-Type Information.One of the most significant limitations is the lack of information regarding cancer diagnoses.Different cancer types have substantially different fall risk profiles due to variations in:• Disease burden;• Functional impairment;• Neurotoxicity;• Treatment side effects.Variables such as breast cancer, lung cancer, gastrointestinal cancer, hematological malignancies, and metastatic disease should be reported and considered in the analysis.

Author response：We thank the reviewer for the valuable comments. We fully agree that cancer type is a key variable influencing fall risk perception, as different cancer types vary substantially in disease burden, functional impairment, neurotoxicity, and treatment-related side effects. The initial design of this study focused on psychosocial factors and did not prespecify cancer type as a core variable. Moreover, the sample sizes for certain cancer types were too small to support stable subgroup analyses. In response, we have added a "Type of tumor" row in Table 1, reporting the counts and percentages of each cancer type. We explicitly acknowledge that cancer type information was not systematically analyzed as a limitation, which has been stated in the Limitations section. Additionally, in the Conclusion section, we recommend that future studies include cancer type as a key covariate and explore the interaction mechanisms between cancer type and fall risk perception.(P8-9 L255; P22 L462-466; P23 L485-486).

#2：Single-Center Convenience Sampling.Participants were recruited from a single tertiary hospital.This introduces potential:•Selection bias•Regional bias•Institutional practice bias.which limits external validity and generalizability.

Author response：We thank the reviewer for the rigorous correction. We fully agree that single-center convenience sampling carries risks of selection bias, regional bias, and institutional practice bias, which limit the generalizability and external validity of the findings. However, the hospital selected for this study is a provincial-level regional medical center in Shandong Province. It currently operates three campuses with approximately 4,000 open beds, including 400 beds in the oncology department, 80 beds in the hematology department, and 35 beds in the radiotherapy department. Its medical services cover multiple surrounding cities, serving a population of approximately 8 million. The hospital admits more than 20,000 cancer patients annually, making it representative in the field of regional oncology diagnosis and treatment. We have revised the Limitations and Conclusion sections accordingly, and all changes are highlighted in red. Thank you again for your valuable comment.(P22 L450-456).

#3：Incomplete Reporting of Missing Data. The manuscript states that multiple imputation was performed; however, critical information is missing:•Percentage of missing data•Missing data mechanism•Number of imputations•Imputation procedures.This information is necessary for reproducibility and transparency.

Author response：We thank the reviewer for the rigorous correction. Regarding the issue of incomplete reporting of missing data, we have carefully verified each item and respond as follows:A total of 560 questionnaires were distributed in this study. Following rigorous quality control, 26 invalid responses were excluded based on the inclusion and exclusion criteria: 1) contradictory answers (n=15): logically inconsistent responses; 2) patterned responses (n=8): overly uniform response patterns; and 3) short completion time (n=3): completion time of less than 3 minutes, far below the average completion time. The final valid sample size was 534 cases, and all study variables had no missing values in the dataset ultimately included for analysis.The statement "Missing data were handled using multiple imputation" in the original manuscript was a writing error and did not reflect the actual data processing procedure. We sincerely apologize for this mistake and have corrected it in the revised manuscript.(P7 L223-225).

#4：Lack of Multicollinearity Assessment.Several psychosocial variables included in the regression model are conceptually correlated, including:•Health literacy•Self-efficacy•Social support.The authors should report:•Variance Inflation Factors (VIF)•Tolerance values•Collinearity diagnostics to demonstrate model stability.

Author response：Thank you for raising this important methodological issue. Your concern regarding potential multicollinearity among psychosocial variables such as health literacy, self-efficacy, and social support is very reasonable. As requested, we conducted collinearity diagnostics for all independent variables included in the regression model. The results showed: health literacy (VIF=1.129, Tolerance=0.886); self-efficacy (VIF=1.127, Tolerance=0.888); social support (VIF=1.082, Tolerance=0.924). All VIF values were < 5 and all Tolerance values were > 0.2. The results have been added to Table 5 (see Table 5 for details).(P8 L242-245; P14 L299-302;P16-17 L310-311).

#5：Limited Reporting of LPA Classification Quality.Although AIC, BIC, entropy, LMRT, and BLRT were reported, additional classification quality indices are recommended: Classification accuracy for each latent class.

Author response：Thank you for this professional and important suggestion. We fully agree that reporting only the overall Entropy is insufficient for comprehensively evaluating the classification quality of LPA, and that reporting classification accuracy for each latent class is essential for verifying the reliability and interpretability of the model. We extracted the average posterior probabilities from the Mplus output file as the core indicator for evaluating classification accuracy for each latent class. The average posterior probabilities for the three latent classes were 1.000, 1.000, and 0.993, respectively, all significantly higher than the recommended threshold of 0.80, indicating that the LPA model in this study has excellent classification accuracy with good distinction among latent classes. The classification accuracy for each latent class has now been added to Table 2, and all revisions have been highlighted in red in the revised manuscript. Thank you again for your rigorous scrutiny of LPA methodological quality.(P8 L236-238; P11 L266-269; P12 L284).

#6：Cross-Sectional Design.The cross-sectional nature of the study precludes causal inference.Terms such as“influencing factors”and“predictors”should be used cautiously and replaced with“associated factors”where appropriate.

Author response：Thank you for pointing out this important issue. We fully agree that the cross-sectional design precludes causal inference, and the use of causally suggestive language could mislead readers. We have systematically reviewed and revised the entire manuscript accordingly, and all changes have been highlighted in red in the revised manuscript.(P1 L20; P2 L42; P4 L106 L112; P5 L148).

Areas for Improvement

#7：Language Quality.Numerous grammatical and stylistic issues are present throughout the manuscript, including:•Inconsistent tense usage•Missing articles•Subject-verb disagreement•Non-native English phrasing.Professional language editing is strongly recommended.

Author response：Thank you for pointing out the language quality issues. We have commissioned a professional team to systematically edit the entire manuscript, correcting problems such as inconsistent tense usage, missing articles, subject-verb disagreement, and non-native English phrasing. All are marked in red.

#8：Data Consistency.There appears to be inconsistency regarding the reported sex distribution of participants between the Results section and Table 1.（line236 Results:

“including 246 (51.90%) 236men and 228 (48.10%) women.”；line238：Table 1:Male=46.1%）Female 288 (53.9%)…The authors should carefully verify all reported statistics.

Author response：Thank you very much for pointing out this data inconsistency issue. We immediately verified all raw data and the numbers reported in the manuscript item by item. Upon verification, we confirmed that a data inconsistency did exist. This error has been corrected in the sex distribution data in the Results section. We sincerely apologize for this oversight in data entry and proofreading. We have now rechecked all data in the manuscript and confirmed that no other errors exist. We apologize again and thank you for your rigorous review.(P8 L253).

#9：Overly Lengthy Discussion.The Discussion section could be reduced by approximately 20-30% to improve focus and readability.

Author response：Thank you for your comment. We have streamlined the Discussion section as requested, primarily by removing redundant statements, excessive modifiers, and overly extended content. All revisions have been highlighted in red in the revised manuscript.(P18-19 L325-350; P19 L352-354; P19-21 L373-443).

Attachment Submitted filename: Response to Reviewers.pdf

# Editor report: Decision Letter 1

**DOI:** 10.1371/journal.pone.0358845.r003

**Authors:** Mary Diane Clark

17 Aug 2026

Dear Dr. Zhang,

plosone@plos.org. When you're ready to submit your revision, log on to https://www.editorialmanager.com/pone/ and select the 'Submissions Needing Revision' folder to locate your manuscript file.

- A letter that responds to each point raised by the academic editor and reviewer(s). You should upload this letter as a separate file labeled 'Response to Reviewers'.
- A marked-up copy of your manuscript that highlights changes made to the original version. You should upload this as a separate file labeled 'Revised Manuscript with Track Changes'.
- An unmarked version of your revised paper without tracked changes. You should upload this as a separate file labeled 'Manuscript'.

If you would like to make changes to your financial disclosure, please include your updated statement in your cover letter. Guidelines for resubmitting your figure files are available below the reviewer comments at the end of this letter.

If applicable, we recommend that you deposit your laboratory protocols in protocols.io to enhance the reproducibility of your results. Protocols.io assigns your protocol its own identifier (DOI) so that it can be cited independently in the future. For instructions see: https://journals.plos.org/plosone/s/submission-guidelines#loc-laboratory-protocols. Additionally, PLOS ONE offers an option for publishing peer-reviewed Lab Protocol articles, which describe protocols hosted on protocols.io. Read more information on sharing protocols at https://plos.org/protocols?utm_medium=editorial-email&utm_source=authorletters&utm_campaign=protocols.

As the corresponding author, your ORCID iD is verified in the submission system and will appear in the published article. PLOS supports the use of ORCID, and we encourage all coauthors to register for an ORCID iD and use it as well. Please encourage your coauthors to verify their ORCID iD within the submission system before final acceptance, as unverified ORCID iDs will not appear in the published article. *Only*  the individual author can complete the verification step; PLOS staff *cannot*  verify ORCID iDs on behalf of authors.

We look forward to receiving your revised manuscript.

Kind regards,

Mary Diane Clark, PhD

Academic Editor

PLOS One

Journal Requirements:

If the reviewer comments include a recommendation to cite specific previously published works, please review and evaluate these publications to determine whether they are relevant and should be cited. There is no requirement to cite these works unless the editor has indicated otherwise.

Please review your reference list to ensure that it is complete and correct. If you have cited papers that have been retracted, please include the rationale for doing so in the manuscript text, or remove these references and replace them with relevant current references. Any changes to the reference list should be mentioned in the rebuttal letter that accompanies your revised manuscript. If you need to cite a retracted article, indicate the article’s retracted status in the References list and also include a citation and full reference for the retraction notice.

**Additional Editor Comments:**

Thank you for the excellent revisions. The M+ is clear and the tables are effective in your results and the discussion is effective.

There are a few things you need to address:All of these measures should be headings and if they are APA level 4 then they are wrong but I am not sure what format you are using

(1)General information questionnaire:

You have a general list here. So it is not a sentence or a paragraph

You can make a table, attached the survey as an appendix, or simply say it included X number of questions related to and name a few and then say see Table or Appendix

(4)The Health Literacy Management Scale was originally developed by Jordan et al. [35] and the was adapted into Chinese by Sun Haolin in 2012 [36].

Shuiyuan [34] in 1986, is was used to

You need to set up a at analytic section and explicitly name the variables and what analyzes you will do

The M+_ analysis area great

But the demographic data goes into the participant section

Thank you for this wonderful paper

[Note: HTML markup is below. Please do not edit.]

[NOTE: If reviewer comments were submitted as an attachment file, they will be attached to this email and accessible via the submission site. Please log into your account, locate the manuscript record, and check for the action link "View Attachments". If this link does not appear, there are no attachment files.]

To ensure your figures meet our technical requirements, please review our figure guidelines: https://journals.plos.org/plosone/s/figures

You may also use PLOS’s free figure tool, NAAS, to help you prepare publication quality figures: https://journals.plos.org/plosone/s/figures#loc-tools-for-figure-preparation.

NAAS will assess whether your figures meet our technical requirements by comparing each figure against our figure specifications.

# Author comment: Author response to Decision Letter 2

**DOI:** 10.1371/journal.pone.0358845.r004

28 Aug 2026

Response to reviewers

Dear reviewers：

Thank you very much for reading this article in your busy schedule, and thank the editorial team and reviewers for their feedback on our manuscript, and provide many constructive suggestions for revision, which have greatly helped us to further improve the article. We have carefully considered and analyzed the opinions of peer reviewers, and on this basis, we have revised the articles one by one.

To reviewer’ comments

#1："All of these measures should be headings and if they are APA level 4 then they are wrong but I am not sure what format you are using"

Author response：We thank the reviewer for this valuable suggestion. We have revised the headings of all measures in the Methods section. Each scale (General Information Questionnaire, Fall Risk Perception Scale, Social Support Rating Scale, Health Literacy Management Scale, and Self-Efficacy to Manage Chronic Disease Scale) is now presented as an independent Level 3 heading (left-aligned and bold), in accordance with the journal's formatting requirements. The revisions have been highlighted in the revised manuscript.(P6 L175, L186, L196;P7 L207).

#2："You have a general list here. So it is not a sentence or a paragraph. You can make a table, attached the survey as an appendix, or simply say it included X number of questions related to and name a few and then say see Table or Appendix".

Author response：We agree with the reviewer that listing all general information items in the text is inappropriate. We have revised this section to a single sentence (General demographic information includes 23 variables, such as gender and age; for detailed information, please refer to the appendix., as suggested. The revision has been highlighted in the manuscript.(P5 L164-166).

#3：The Health Literacy Management Scale was originally developed by Jordan et al. [35] and the was adapted into Chinese by Sun Haolin in 2012 [36]. Shuiyuan [34] in 1986, is was used to.

Author response：We thank the reviewer for the rigorous correction. Thank you very much for pointing out the grammatical and expressive errors in the scale citation section; we sincerely apologize for these oversights resulting from carelessness. The revised sections have been marked in red.(P6 L187-188,197-200).

#4：You need to set up a at analytic section and explicitly name the variables and what analyzes you will do. The M+ analysis area great. But the demographic data goes into the participant section.

Author response：Thank you very much for your guidance on the statistical analysis section. It should be noted that we have already clearly reported in the "Statistical Analysis" section the statistical software used in this study (Mplus 8.3 and SPSS 26.0), the analytical methods applied to each variable, the metrics for evaluating the fit of the LPA model, the diagnostic criteria for multicollinearity, and the significance levels. Additionally, the descriptive statistics for demographic data have been presented, as required, in the "Demographic and Clinical Characteristics" subsection at the beginning of the "Results" section (Table 1); they are not repeated in the Statistical Analysis section. General information is provided in the dedicated Participant Section within the Study Design.(P5 L164-166; P8 L245-260).

Attachment Submitted filename: Response to Reviewers.docx

# Editor report: Decision Letter 2

**DOI:** 10.1371/journal.pone.0358845.r005

**Authors:** Mary Diane Clark

2 Sep 2026

Dear Dr. Zhang,

Please submit your revised manuscript by Oct 17 2026 11:59PM. If you will need more time than this to complete your revisions, please reply to this message or contact the journal office at plosone@plos.org. When you're ready to submit your revision, log on to https://www.editorialmanager.com/pone/ and select the 'Submissions Needing Revision' folder to locate your manuscript file.

- A letter that responds to each point raised by the academic editor and reviewer(s). You should upload this letter as a separate file labeled 'Response to Reviewers'.
- A marked-up copy of your manuscript that highlights changes made to the original version. You should upload this as a separate file labeled 'Revised Manuscript with Track Changes'.
- An unmarked version of your revised paper without tracked changes. You should upload this as a separate file labeled 'Manuscript'.

If you would like to make changes to your financial disclosure, please include your updated statement in your cover letter. Guidelines for resubmitting your figure files are available below the reviewer comments at the end of this letter.

If applicable, we recommend that you deposit your laboratory protocols in protocols.io to enhance the reproducibility of your results. Protocols.io assigns your protocol its own identifier (DOI) so that it can be cited independently in the future. For instructions see: https://journals.plos.org/plosone/s/submission-guidelines#loc-laboratory-protocols. Additionally, PLOS One offers an option for publishing peer-reviewed Lab Protocol articles, which describe protocols hosted on protocols.io. Read more information on sharing protocols at https://plos.org/protocols?utm_medium=editorial-email&utm_source=authorletters&utm_campaign=protocols.

As the corresponding author, your ORCID iD is verified in the submission system and will appear in the published article. PLOS supports the use of ORCID, and we encourage all coauthors to register for an ORCID iD and use it as well. Please encourage your coauthors to verify their ORCID iD within the submission system before final acceptance, as unverified ORCID iDs will not appear in the published article. *Only*  the individual author can complete the verification step; PLOS staff *cannot*  verify ORCID iDs on behalf of authors.

We look forward to receiving your revised manuscript.

Kind regards,

Mary Diane Clark, PhD

Academic Editor

PLOS One

**Journal Requirements:**

1. If the reviewer comments include a recommendation to cite specific previously published works, please review and evaluate these publications to determine whether they are relevant and should be cited. There is no requirement to cite these works unless the editor has indicated otherwise.

2. Please review your reference list to ensure that it is complete and correct. If you have cited papers that have been retracted, please include the rationale for doing so in the manuscript text, or remove these references and replace them with relevant current references. Any changes to the reference list should be mentioned in the rebuttal letter that accompanies your revised manuscript. If you need to cite a retracted article, indicate the article’s retracted status in the References list and also include a citation and full reference for the retraction notice.

**Additional Editor Comments:**

Thank you for taking care of all but one comment from the last review. You added Participants at 164 with a statement of looking towards the table that is still in the results. Partiipant data goes up into the method---please move it up to that point..

Then eliminate the heading at line 262

Finally, on 302 you have a hanging or orphan heading. Please use a hard page break to force it down to the page below.

Sorry to ask for. one more change but these should take about 1/2 hour and then I will accept it.

[Note: HTML markup is below. Please do not edit.]

[NOTE: If reviewer comments were submitted as an attachment file, they will be attached to this email and accessible via the submission site. Please log into your account, locate the manuscript record, and check for the action link "View Attachments". If this link does not appear, there are no attachment files.]

To ensure your figures meet our technical requirements, please review our figure guidelines: https://journals.plos.org/plosone/s/figures

You may also use PLOS’s free figure tool, NAAS, to help you prepare publication quality figures: https://journals.plos.org/plosone/s/figures#loc-tools-for-figure-preparation.

NAAS will assess whether your figures meet our technical requirements by comparing each figure against our figure specifications.

# Author comment: Author response to Decision Letter 3

**DOI:** 10.1371/journal.pone.0358845.r006

6 Sep 2026

To reviewer’ comments

#1："You added Participants at 164 with a statement of looking towards the table that is still in the results. Participant data goes up into the method---please move it up to that point."

Author response：We thank the editor for this valuable suggestion. We have moved the descriptive participant data to the Methods section under the “Participants” subsection (around line 164) , while retaining the reference to the relevant table. (P4 L164).

#2：The redundant heading at line 262 should be removed.

Author response：We have deleted that heading as requested and checked the surrounding text to ensure no loss of coherence.

#3：The heading at line 302 appears as an orphan/widow heading and should be forced to the next page.

Author response：We thank the editor for the rigorous correction. We have inserted a hard page break before that heading, ensuring it now appears at the top of the following page, in compliance with the journal’s formatting requirements.(P12 L303).

Attachment Submitted filename: Response_to_Reviewers_auresp_3.docx

# Editor report: Decision Letter 3

**DOI:** 10.1371/journal.pone.0358845.r007

**Authors:** Mary Diane Clark

7 Sep 2026

Perception of fall risk among cancer survivors receiving Radiotherapy/Chemotherapy in China：A latent profile analysis

PONE-D-26-19655R3

Dear Dr. Zhang,

We’re pleased to inform you that your manuscript has been judged scientifically suitable for publication and will be formally accepted for publication once it meets all outstanding technical requirements.

Within one week, you’ll receive an e-mail detailing the required amendments. When these have been addressed, you’ll receive a formal acceptance letter and your manuscript will be scheduled for publication.

An invoice will be generated when your article is formally accepted. Please note, if your institution has a publishing partnership with PLOS and your article meets the relevant criteria, all or part of your publication costs will be covered. Please make sure your user information is up-to-date by logging into Editorial Manager at Editorial Manager® and clicking the ‘Update My Information' link at the top of the page. For questions related to billing, please contact billing support.

If your institution or institutions have a press office, please notify them about your upcoming paper to help maximize its impact. If they’ll be preparing press materials, please inform our press team as soon as possible -- no later than 48 hours after receiving the formal acceptance. Your manuscript will remain under strict press embargo until 2 pm Eastern Time on the date of publication. For more information, please contact onepress@plos.org.

Kind regards,

Mary Diane Clark, PhD

Academic Editor

PLOS One

Additional Editor Comments (optional):

Thank you for making those few last corrections. Congrats on an interesting paper.

Reviewers' comments:

# Editor report: Acceptance letter

**DOI:** 10.1371/journal.pone.0358845.r008

**Authors:** Mary Diane Clark

PONE-D-26-19655R3

PLOS One

Dear Dr. Zhang,

I'm pleased to inform you that your manuscript has been deemed suitable for publication in PLOS One. Congratulations! Your manuscript is now being handed over to our production team.

At this stage, our production department will prepare your paper for publication. This includes ensuring the following:

* All references, tables, and figures are properly cited

* All relevant supporting information is included in the manuscript submission,

* There are no issues that prevent the paper from being properly typeset

You will receive further instructions from the production team, including instructions on how to review your proof when it is ready. Please keep in mind that we are working through a large volume of accepted articles, so please give us a few days to review your paper and let you know the next and final steps.

Lastly, if your institution or institutions have a press office, please let them know about your upcoming paper now to help maximize its impact. If they'll be preparing press materials, please inform our press team within the next 48 hours. Your manuscript will remain under strict press embargo until 2 pm Eastern Time on the date of publication. For more information, please contact onepress@plos.org.

You will receive an invoice from PLOS for your publication fee after your manuscript has reached the completed accept phase. If you receive an email requesting payment before acceptance or for any other service, this may be a phishing scheme. Learn how to identify phishing emails and protect your accounts at https://explore.plos.org/phishing.

If we can help with anything else, please email us at customercare@plos.org.

Thank you for submitting your work to PLOS One and supporting open access.

Kind regards,

PLOS One Editorial Office Staff

on behalf of

Dr. Mary Diane Clark

Academic Editor

PLOS One
